# Comparative genomic analysis reveals contraction of gene families with putative roles in pathogenesis in the fungal boxwood pathogens *Calonectria henricotiae* and *C. pseudonaviculata*

**DOI:** 10.1186/s12862-022-02035-4

**Published:** 2022-06-21

**Authors:** Layne W. Rogers, Alyssa M. Koehler, Jo Anne Crouch, Marc A. Cubeta, Nicholas R. LeBlanc

**Affiliations:** 1 Department of Entomology and Plant Pathology, North Carolina State University, Center for Integrated Fungal Research, Raleigh, NC 27695 USA; 2 Department of Plant and Soil Sciences, University of Delaware, Georgetown, DE 19947 USA; 3 United States Department of Agriculture–Agricultural Research Service, Mycology and Nematology Genetic Diversity and Biology Laboratory, Beltsville, MD 20705 USA; 4 United States Department of Agriculture–Agricultural Research Service, Crop Improvement and Protection Research Unit, 1636 E. Alisal St., Salinas, CA 93905 USA

**Keywords:** Nectriaceae, Host range, Trophic lifestyle

## Abstract

**Background:**

Boxwood blight disease caused by *Calonectria henricotiae* and *C. pseudonaviculata* is of ecological and economic significance in cultivated and native ecosystems worldwide. Prior research has focused on understanding the population genetic and genomic diversity of *C. henricotiae* and *C. pseudonaviculata*, but gene family evolution in the context of host adaptation, plant pathogenesis, and trophic lifestyle is poorly understood. This study applied bioinformatic and phylogenetic methods to examine gene family evolution in *C. henricotiae*, *C. pseudonaviculata* and 22 related fungi in the Nectriaceae that vary in pathogenic and saprobic (apathogenic) lifestyles.

**Results:**

A total of 19,750 gene families were identified in the 24 genomes, of which 422 were rapidly evolving. Among the six *Calonectria* species, *C. henricotiae* and *C. pseudonaviculata* were the only species to experience high levels of rapid contraction of pathogenesis-related gene families (89% and 78%, respectively). In contrast, saprobic species *Calonectria multiphialidica* and *C. naviculata*, two of the closest known relatives of *C. henricotiae* and *C. pseudonaviculata*, showed rapid expansion of pathogenesis-related gene families.

**Conclusions:**

Our results provide novel insight into gene family evolution within *C. henricotiae* and *C. pseudonaviculata* and suggest gene family contraction may have contributed to limited host-range expansion of these pathogens within the plant family Buxaceae.

**Supplementary Information:**

The online version contains supplementary material available at 10.1186/s12862-022-02035-4.

## Background

Boxwood blight is an emerging invasive disease of broadleaf evergreen shrubs and trees in the plant family Buxaceae [15]. Due to the widespread commercial production and negative impact of boxwood blight in native ecosystems, this disease poses a major threat to the worldwide ornamental horticulture industry and native *Buxus* populations in Asia and Europe [47]. Boxwood blight was first discovered in the United Kingdom in 1994 and subsequently identified in the United States in 2011, where it occurs in 30 states and the District of Columbia [27, 29, 32]. Symptoms of boxwood blight begin as dark lesions on leaves of infected plants that eventually lead to extreme leaf blighting, stem lesions, and defoliation under conducive environmental conditions. Few effective boxwood disease management practices have been identified and current research efforts are focused on developing host–plant resistance strategies as multiple fungicide applications are costly and unsustainable for the year-round occurrence of this disease [15].

Boxwood blight is caused by the ascomycete fungi *Calonectria henricotiae* (*Che*) and *C. pseudonaviculata* (*Cps*), two closely related sister species in the family Nectriaceae. *Cps* also infects two additional genera in the plant family Buxaceae, *Pachysandra* and *Sarcococca* [7]. When boxwood blight disease was first discovered in the 1990s in Europe, *Cps* was the only known causal agent. However, in 2005, a second species—*Che* was identified from diseased boxwood in the UK and continental Europe [24]. Several studies have shown limited genetic diversity from natural populations of *Che* and *Cps*, consistent with the hypothesis of predominant asexual reproduction and introduced clonal lineages [8, 37, 38]. Malapi-Wight et al. (2019) determined that populations of *Cps* have a single mating type idiomorph (MAT1-1) compared to populations of *Che* which possess the MAT1-2 idiomorph [44]. Separate studies demonstrated that pathogen populations have low genetic diversity and no evidence of sexual recombination, suggesting limited opportunities for mating with predominately clonal asexual reproduction [8, 37]. Despite possessing opposite mating types and a sympatric geographic distribution in Europe and the UK, successful mating between *Che* and *Cps* has not been observed in nature or under laboratory conditions [7, 37, 44]. Additional population genomic analyses of *Che* and *Cps* have also shown limited gene flow between the two species and absence of shared genetic polymorphisms [38].

The genus *Calonectria* includes more than 160 described species that inhabit a broad range of ecological habitats and lifestyles globally [41]. In addition to *Che* and *Cps*, several other species of *Calonectria* are plant pathogens and the causal agents of diseases on approximately 335 plant species across 100 plant families [13, 42]*.* For example, *C. ilicicola* infects at least 70 plant species in multiple families while *C. gordoniae* is a pathogen of a single host plant species native to the southeastern US, loblolly bay (*Gordonia lasianthus*) [21]. Despite the incredible diversity of lifestyles employed by *Calonectria* species, little is known about the mechanisms that these fungi utilize to successfully infect their hosts and extract nutrients. Two comparative genomic and transcriptomic studies were recently conducted on the *Eucalyptus* pathogen *C. pseudoreteaudii* (*Cpr*) to elucidate pathogenesis mechanisms. In these studies, enzymes involved in secondary (specialized) metabolite biosynthesis were up-regulated in *Cpr* mycelia grown in *Eucalyptus* tissue culture medium [75]. These authors identified expanded gene families of Major Facilitator Superfamily (MFS) transporters that enhance pathogenicity suggesting that MFS proteins may provide an adaptive mechanism for degrading and transporting compounds produced by *Eucalyptus* that are toxic to the fungus. Ye et al. (2017) analyzed *Cpr* gene expression profiles at three temporal stages of *Eucalyptus* infection and disease symptom development. The authors identified differentially expressed genes involved in plant cell wall degradation, detoxification of phytoalexins, toxin synthesis, iron uptake, and reactive oxygen species scavenging. Genes encoding cutinase enzymes, which are crucial for plant pathogenic fungi that penetrate through the host cuticle, were also up-regulated during plant pathogenesis and expressed earlier than other cell wall degrading enzymes [76]. An additional report of secondary metabolites as virulence factors was observed in *C. iliciola* and production of the PF1070A phytotoxin was correlated with increased disease symptom expression in 17 isolates examined [49]. The extracellular proteomes of *Che* and *Cps* were recently examined and revealed 124 putative effectors produced by both species which are hypothesized to be involved in plant pathogenesis [74]. However, to date, gene expression profiles of *Che* and *Cps* during boxwood blight disease development have not been the subject of comprehensive investigation in a gene family evolution framework.

During genome evolution, gene duplication and gene loss events can contribute to contraction and expansion of gene families [17]. Genome changes can be linked to evolutionary processes that result in environmental niche adaptation [17]. Studying changes in gene family contraction and expansion can provide useful insight into organismal, ecological, and lifestyle transitions of plant pathogenic fungi. In many disease-causing fungal species, changes in gene family size have been linked to observed variation in host adaptation, pathogenesis, and virulence [54, 61]. Rapid expansion of gene families in plant pathogenic fungi associated with host cell wall degradation, secondary, and carbohydrate metabolism is providing insight into pathogenesis and virulence processes [40, 48, 64]. Rapid contraction of gene families involved in similar processes have also been linked to biotrophy (obligate parasitism) and ecological lifestyle [65, 78]. For example, in insect and plant pathogenic fungi, contraction of gene families was associated with cuticle and cell wall degradation and limited (narrow) host range [3, 71]. In plant and insect systems, analysis of gene family evolution has elucidated different aspects of pathogen biology and ecology. In the Northern California black walnut (*Juglans hindsii*), a plant species native to the western US, contraction of gene families involved in abiotic stress and disease were associated with resistance to Armillaria root rot disease [69]. In another recent study, identification of rapidly evolving gene families led to the development of novel strategies for managing blood-feeding insects [23].

In this study, we deployed comparative phylogenomic tools to characterize and identify rapidly evolving gene families within the genomes of *Che*,* Cps*, and 22 additional fungal taxa. Using multiple analytical methods, we generated annotations for protein sequences within rapidly evolving gene families to determine putative functional classes. Further annotation of putative pathogenicity factors and secreted effectors within rapidly evolving gene families of *Che*, *Cps*, closely related plant pathogenic and saprobic (apathogenic) species of *Calonectria*, and less-aggressive pathogens of hosts in Buxaceae *Pseudonectria buxi* (*Pbu*),* P. foliicola* (*Pfo*), and *Coccinonectria pachysandricola* (*Cpa*), were conducted to identify shared patterns in gene family evolution associated with fungal-host plant adaptation, pathogenesis, and virulence. We hypothesized that gene families important for host infection and pathogenesis have expanded in *Che* and *Cps*, relative to other pathogenic and saprobic species of *Calonectria* and closely related non-*Calonectria* Buxaceae pathogens. Here we report on (1) the quantity and predicted functional classes of rapidly contracting and expanding gene families in 24 fungal taxa in the Nectriaceae that vary in pathogenic and saprobic lifestyle; and (2) the predicted functional annotation and comparison of putative pathogenicity factors and secreted effectors within rapidly evolving gene families of *Che*, *Cps*, and closely and distantly related species of *Calonectria* and non-*Calonectria* pathogens of hosts in Buxaceae.

## Results

### Gene family identification based on time-calibrated phylogenetic analyses

Genome assemblies and predicted proteomes for each of the 24 fungal taxa showed high levels of completeness based on BUSCO scores of 95% or higher (Additional file 1). Overall, 95% of the predicted protein sequences across all the taxa were assigned to a gene family, with a total of 19,750 gene families identified. The average number of proteins in a gene family was 16.7 and 2154 gene families had single copy proteins found in all 24 taxa. Construction of a maximum likelihood phylogenetic tree using protein sequence data from 2154 single copy genes showed 100% confidence in tree topology (Additional file 2).

### Identification of rapidly evolving gene families

**Fig. 1:**
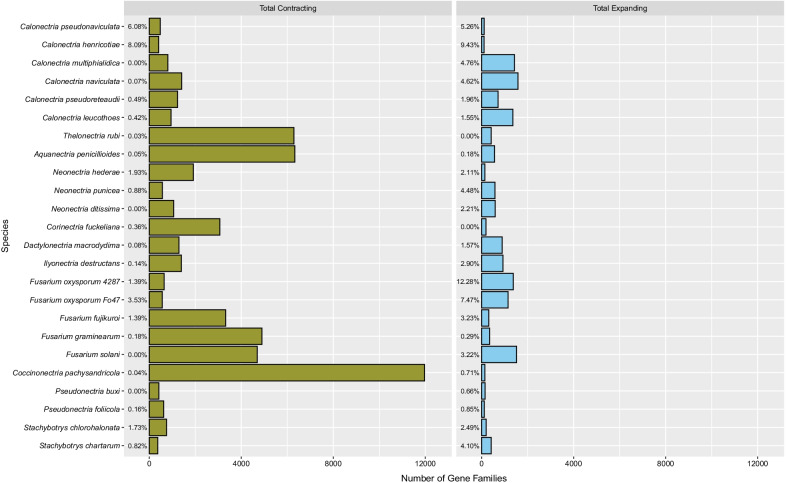
Number of total evolving gene families for the boxwood blight pathogens *Calonectria henricotiae* and *C. pseudonaviculata*, and 22 fungal taxa in the Nectriaceae. Percentages to the left of the bars represent the percent of total changing gene families that are rapidly evolving

Across the time-calibrated phylogeny of the 24 fungal taxa examined in this study, CAFE4 identified 422 gene families evolving at a non-random rate (rapidly evolving) (p ≤ 0.01; Additional file 3, Additional file 4). In total, 17,596 gene families experienced a change in size across the phylogeny, either through expansion or contraction. To provide a measure of rapid gene family evolution that each species experienced relative to total changing (both rapidly evolving and randomly evolving) gene families, calculations of the percent of rapidly evolving gene families per total changing gene families were performed (Fig. 1). For 17 species, rapidly expanding gene families accounted for ≥ 1% of total expanding gene families. Rapidly contracting gene families accounted for ≥ 1% of total contracting gene families in seven species. However, randomly contracting gene families were more numerous than randomly expanding gene families across the 24 taxa and may partially explain the generally lower observed percentages.

**Fig. 2:**
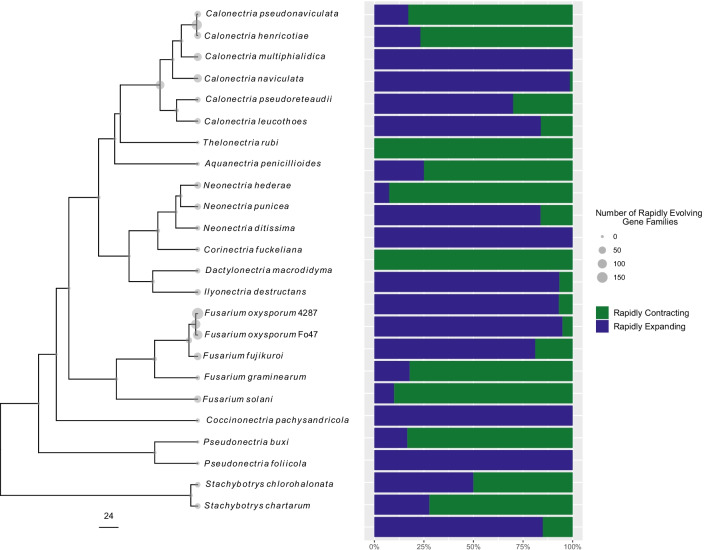
Time-calibrated maximum likelihood tree constructed from 2154 single copy orthologs that showed 100% confidence in tree topology for the boxwood blight pathogens *Calonectria henricotiae* and *C. pseudonaviculata*, and 22 fungal taxa in the Nectriaceae. The scale bar units are in millions of years. Percentages and frequencies of rapidly expanding and contracting gene families are plotted to the right of the phylogeny

The percentage of rapidly evolving gene families showed variation among the 24 fungal taxa. For example, *Cpa*, which has the smallest assembled genome among the 24 taxa (26.4 Mb), contained the largest number of total contracting gene families (11,967 gene families) and mean gene losses (-8.4 genes) but was one of five species to have < 10 total rapidly evolving gene families (six gene families) (Fig. 1, Additional file 1, and Additional file 5). Surprisingly, *Che* exhibited the highest and second highest percentages of rapidly contracting and expanding (respectively) gene families, despite having the second and first smallest totals (contracting and expanding, respectively) (Fig. 1). Similar to *Che*,* Cps* experienced comparable trends in both total changing and total rapidly evolving gene families (Fig. 1 and Additional file 5). The proportion of rapidly expanding gene families compared to rapidly contracting gene families for each species showed that each of the 24 taxa exhibited distinct patterns of gene family evolution directionality, with either more rapid gene family contraction or more rapid gene family expansion (Fig. 2).

Among species of *Calonectria*, *Che* and *Cps* were the only species to undergo more rapid gene family contractions than expansions and had considerably fewer total expanding and contracting gene families than *Cmu*, *Cna*,* Cle*, and *Cpr* (Figs. 1, 2, and Additional file 5). The saprobic species *Cmu* and *Cna* exhibited nearly exclusive rapid gene family expansion. For example, *Cna* had the greatest number of total expanding gene families across all 24 taxa (Figs. 1, 2, and Additional file 5). Compared to the non-*Calonectria* Buxaceae pathogens (*Cpa*,* Pbu*, and *Pfo*), *Che* and *Cps* experienced more rapid gene family expansions and contractions than *Cpa*,* Pbu*, and *Pfo*. However, *Che* and *Cps* had a similar number of total contracting and expanding gene families compared to the two species of *Pseudonectria* examined in this study. For example, *Pseudonectria* species had ~ 11,000 fewer total contracting gene families than *Cpa*, which had the most total contracting gene families among the 24 taxa examined (Figs. 1, 2, and Additional file 5). The non-*Calonectria* pathogens of plants in the family Buxaceae consistently placed in the bottom five species with the fewest total rapidly evolving gene families (*Pbu*,* Pfo*, and *Cpa* had one, two and six total rapidly evolving gene families, respectively; Additional file 5).

To identify rapidly evolving gene families shared between *Che* and *Cps* and the other *Calonectria* species and non-*Calonectria* pathogens of species in Buxaceae (*Cpa*,* Pbu*, and *Pfo*), a series of UpSet plots were generated (Additional file 6). For rapidly contracting and expanding gene families, *Che* and *Cps* shared two or fewer gene families with saprobic *Calonectria* species, pathogenic *Calonectria* species, and non-*Calonectria* pathogens of plants in the family Buxaceae (Additional file 6). *Che* and *Cps* shared the most rapidly contracting and rapidly expanding gene families with the pathogenic *Calonectria* species (*Cle* and *Cp*) (Additional file 6). *Che* and *Cps* did not share any rapidly evolving gene families within the “rapidly expanding” or “rapidly contracting” categories but did share three gene families that were rapidly evolving in opposite directions in each species (OG0000649, OG0001150, and OG0007608). Individually, *Che* and *Cps* experienced rapid expansion and rapid contraction of three gene families (OG0000150, OG0000440, and OG0000796 in *Che*, and OG0000026, OG0000101, and OG0000854 in *Cps*) that were not rapidly evolving in any of the other 22 additional fungal taxa.

### Annotation of rapidly evolving gene families

While 422 gene families were identified by CAFE4 as rapidly evolving across the phylogeny, only those rapidly evolving at terminal taxa were characterized (403 gene families). Of the 7221 protein sequences grouped into the 403 extant gene families, 5912 received a COG annotation (sequences that received an ‘NA’ or not annotated designation by eggNOG were removed from subsequent analyses) (Table 1). The 5912 annotated sequences were used to classify 332 rapidly evolving gene families into a putative functional category which were annotated with between one and 11 (1.56 ± 1.09; Mean ± SD) COG categories based on the annotations of protein sequences within each gene family. Approximately 68% (225 gene families) of the COG-annotated gene families were annotated with a single COG category. For Pfam annotation, 5294 of the 7221 protein sequences received a Pfam hit with an e-value ≤ 1e−5 and spanned 317 of the 403 rapidly evolving gene families with between one and 207 (16.700 ± 22.616) sequences per gene family. Protein sequences that received both a COG and Pfam annotation (4468 out of the 7221 protein sequences) spanned 304 of the 403 rapidly evolving gene families in extant species.

**Table 1:** Number of rapidly evolving gene families and proteins assigned to each Clusters of Orthologous Groups (COG) category across the genomes of the boxwood blight pathogens *Calonectria henricotiae* and *C. pseudonaviculata*, and 22 fungal taxa in the Nectriaceae

COG abbreviation	COG category	Number of rapidly evolving gene families	Number of proteins
S	Function unknown	101	1566
Q	Secondary metabolite biosynthesis, transport, and catabolism	40	709
G	Carbohydrate transport and metabolism	19	240
E	Amino acid transport and metabolism	17	228
C	Energy production and conversion	16	207
K	Transcription	16	229
L	Replication, recombination, and repair	12	114
T	Signal transduction mechanisms	12	118
I	Lipid transport and metabolism	8	118
P	Inorganic ion transport and metabolism	8	99
U	Intracellular trafficking, secretion, and vesicular transport	8	105
Z	Cytoskeleton	7	131
M	Cell wall/membrane/envelope biogenesis	6	39
O	Post-translational modification, protein turnover, chaperones	6	252
B	Chromatin structure and dynamics	4	45
H	Coenzyme transport and metabolism	3	9
J	Translation, ribosomal structure, and biogenesis	3	37
V	Defense mechanisms	3	48
A	RNA processing and modification	2	26
D	Cell cycle control, cell division, chromosome partitioning	2	30
OT	Post-translational modification, protein turnover, chaperones/signal transduction mechanisms	2	24
AT	RNA processing and modification/signal transduction mechanisms	1	17
CG	Energy production and conversion/carbohydrate transport and metabolism	1	13
CH	Energy production and conversion/coenzyme transport and metabolism	1	8
DK	Cell cycle control, cell division, chromosome partitioning/transcription	1	15
FQ	Nucleotide transport and metabolism/secondary metabolite biosynthesis, transport, and catabolism	1	5
IQ	Lipid transport and metabolism/secondary metabolite biosynthesis, transport, and catabolism	1	10
KL	Transcription/replication, recombination, and repair	1	1
TZ	Signal transduction mechanisms/cytoskeleton	1	13
UY	Intracellular trafficking, secretion, and vesicular transport/nuclear structure	1	12

**Fig. 3:**
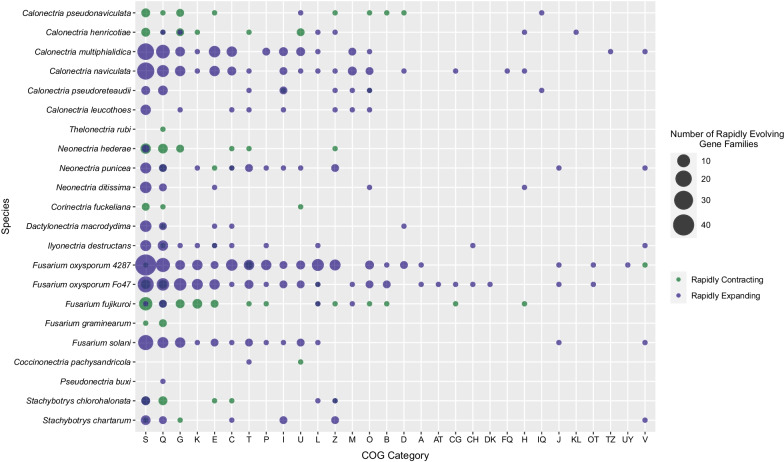
Frequency of annotated gene families in each Cluster of Orthologous Groups (COG) category for the boxwood blight pathogens *Calonectria henricotiae* and *C. pseudonaviculata*, and 22 fungal taxa in the Nectriaceae

Among the 24 fungal taxa, 101 annotated gene families (~ 33%) were of the unknown function COG category (S category; Table 1). Of the 115 Pfam targets (13.6 ± 27.4 proteins per target) spanning the S-categorized gene families, heterokaryon incompatibility protein (HET; PF06985.12; 253 proteins) was the most frequently observed and was found in seven S-categorized gene families. The second most frequently observed Pfam target was NACHT domain (PF05729.13; 97 protein sequences), which is associated with programmed cell death and heterokaryon incompatibility (HET) loci. Together, HET and NACHT domain Pfam targets spanned 13 S-categorized gene families which were rapidly evolving in 14 of the 24 taxa (*Che*,* Cmu*,* Cna*,* Cps*,* Cp*,* Dactylonectria macrodidyma*,* Fusarium fujikuroi*,* F. oxysporum* 4287, *F. oxysporum* Fo47, *F. solani*,* Neonectria hederae*,* N. punicea*,* Stachybotrys chartarum*, and *S. chlorohalonata*) (Fig. 3). Excluding *F. oxysporum* 4287 and Fo47, and *S. chlorohalonata*, each species experienced either exclusive rapid expansion or contraction of these gene families. After S-categorized gene families, Q-categorized gene families (secondary metabolism, biosynthesis, and catabolism) were the second most frequently observed COG category with 40 gene families (709 protein sequences). Pfam targets within Q-categorized gene families spanned a narrower range than in S-categorized gene families of 56 targets (12.7 ± 35.1 proteins per target) with the most frequently observed targets being cytochrome P450 (PF00067.23; 243 protein sequences), short-chain dehydrogenase (PF00106.26; 88 protein sequences), and enoyl-(acyl carrier protein) reductase (PF13561.7; 76 protein sequences). Combined, these Pfam-targets spanned 23 of 40 Q-categorized gene families which were rapidly evolving in 18 of the 24 taxa (*Cp*,* Cmu*,* Cna*,* Cps*,* Corinectria fuckeliana*,* D. macrodidyma*,* F. oxysporum* 4287, *F. fujikuroi*,* F. graminearum*, *F. oxysporum* 47, *F. solani*,* Ilyonectria destructans*,* N. ditissima*,* N. hederae*,* N. punicea*,* Pbu*,* S. chartarum*,* S. chlorohalonata*) (Fig. 3). These gene families were exclusively expanding in half of the species and either both expanding and contracting or exclusively contracting in the remaining species. Combined with the S- and Q-categorized gene families, G-categorized gene families (carbohydrate transport and metabolism; 19 gene families; 240 protein sequences) represented more than 50% of the COG-annotated gene families. Pfam targets within G-categorized gene families spanned 33 Pfam targets (7.27 ± 8.71 proteins per target) with the most frequently observed being major facilitator family (PF07690.17; 39 protein sequences), tannase and feruloyl esterase (PF07519.12; 36 protein sequences), and glycoside hydrolase family 18 (PF00704.29; 15 protein sequences). These three Pfam targets spanned seven of the 19 G-annotated gene families which were rapidly evolving in eight of 24 taxa and exclusively expanding or contracting in each species (*Cmu*,* Cna*,* Cps*,* F. fujikuroi*,* F. oxysporum* Fo47, *F. solani*,* N. hederae*,* S. chartarum*). The remaining 27 COG categories contained ≤ 17 gene families per category and were represented by 304 annotated gene families (Table 1). The comprehensive range of Pfam functional targets within each COG-annotated gene family are presented in Additional file 7.

### Identification of putative pathogenicity factors and secreted effectors

**Fig. 4:**
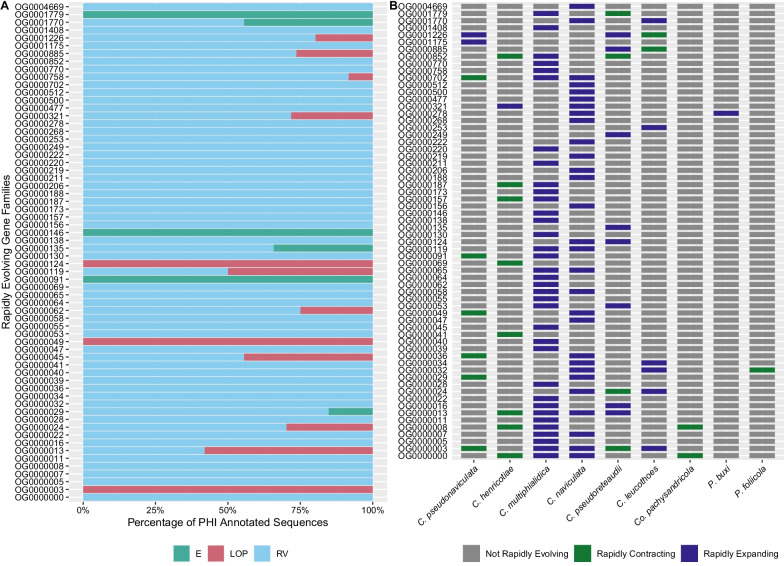
Pathogen host interactions (PHI) annotations for rapidly evolving gene families in the genomes of the boxwood blight pathogens *Calonectria henricotiae* and *C. pseudonaviculata*, saprobic *Calonectria* species, pathogenic *Calonectria* species, and non-*Calonectria* Buxaceae pathogens. **A** Percentage of annotated protein sequences from nine species searched against the PHI database from each rapidly evolving gene family with effector (E), loss of pathogenicity (LOP) and reduced virulence (RV) phenotypes. **B** Gene families containing PHI annotations and direction of rapid evolution (rapid expansion, rapid contraction, not rapidly evolving)

**Fig. 5:**
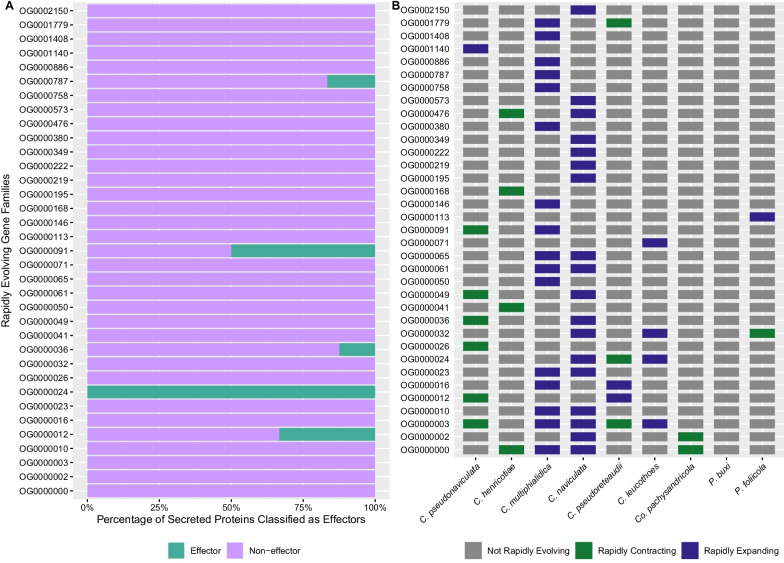
SignalP and EffectorP classifications for rapidly evolving gene families in the boxwood blight pathogens *Calonectria henricotiae* and *C. pseudonaviculata*, saprobic *Calonectria* species, pathogenic *Calonectria* species, and non-*Calonectria* Buxaceae pathogens. **A** Percentage of effector/non-effector proteins from predicted secreted proteins identified by SignalP within each rapidly evolving gene family using classified sequences from each species. **B** Gene families containing secreted proteins and direction of rapid evolution (rapid expansion, rapid contraction, not rapidly evolving)

To further screen rapidly evolving gene families in the genus *Calonectria* and non-*Calonectria* pathogens of hosts in Buxaceae for potential roles in plant pathogenesis, protein sequences in these gene families were compared to accessions in the Pathogen Host Interactions (PHI) database. The PHI database catalogues pathogenicity, virulence, and effector genes that have been experimentally tested in pathogen-host interactions of fungal, oomycete, and bacterial pathogens with animal, plant, fungal, and insect hosts [70]. In total 2682 sequences were searched against the PHI database and 1566 sequences spanning 112 rapidly evolving gene families received hits with e-values ≤ 1e−5. Protein sequences from all species except the non-*Calonectria* Buxaceae pathogen *Pfo* received PHI hits. To identify PHI-annotated sequences putatively involved in virulence and pathogenicity, sequences homologous to proteins with annotated mutant phenotypes of reduced virulence (RV), loss of pathogenicity (LOP), or effector (E) in other pathogens were identified from the dataset. Of 1566 sequences with similarity to sequences in the PHI database, 738 sequences matched these criteria and spanned 64 rapidly evolving gene families (Fig. 4 and Additional file 8). The same set of sequences used for PHI annotation were also classified into secreted and other (no signal peptide identified) protein categories using SignalP v5.0 [1]. Of 2682 sequences, 123 sequences were classified as secreted proteins and spanned 35 rapidly evolving gene families (Fig. 5 and Additional file 8). All species except for *Pbu* and *Cpa* had sequences classified as secreted proteins in rapidly evolving gene families. Sequences classified as secreted proteins were further classified into effector and non-effector categories using EffectorP v2.0 (Fig. 5) [66].

The predominant PHI phenotype for annotated sequences within rapidly evolving gene families was RV followed by LOP and E (Fig. 4 and Additional file 8). Several shared gene families identified with the UpSet plots between *Che*,* Cps*, sapbrobic *Calonectria* species, non-Buxaceae *Calonectria* pathogens, and non-*Calonectria* pathogens of species in Buxaceae also contained PHI-annotated sequences (Fig. 4 and Additional file 6). Of 123 sequences classified as secreted proteins by SignalP, seven were classified as effectors and spanned five gene families which were rapidly expanding in *Cmu*,* Cna*,* Cle*,* and Cpr* and rapidly contracting in *Cps* and *Cpr* (Fig. 5). Gene families containing secreted effectors were not rapidly evolving in *Che.* Gene families rapidly evolving within saprobic *Calonectria* species *Cmu* and *Cna* that contained PHI-annotated and/or predicted secreted effectors experienced exclusive rapid expansion, while gene families rapidly evolving in *Che* and *Cps* that contained sequences with similar annotations experienced predominant rapid contraction (Figs. 4, 5). COG and Pfam annotation information for gene families that contained PHI annotated sequences and/or secreted effectors that were rapidly evolving in *Che* and *Cps* are presented in Table 2. These data showed that 10 gene families belonged to the secondary metabolism (Q), carbohydrate metabolism (G), and intracellular trafficking and secretion (U) COG categories. Among the 10 families, *Che* and *Cps* experienced rapid contraction in eight gene families within these categories (Table 2).

**Table 2:** Rapidly evolving gene families in the genomes of the boxwood blight pathogens *Calonectria henricotiae* (*Che*) and *C. pseudonaviculata* (*Cps*) that contain predicted proteins with pathogen host interactions (PHI) annotations and/or putative secreted effectors

Gene family ID	Rapidly evolving in *Cps*	Rapidly evolving in *Che*	COG category	Top Pfam target	Contains PHI annotated sequences	Contains putative secreted effectors
OG0000000		(C)	S	NACHT (PF05729.13)	Yes	No
OG0000003	(C)		O	CHAT (PF12770.8)	Yes	No
OG0000008		(C)	U	Sugar transporter (PF00083.25)	Yes	No
OG0000012	(C)		Z	Phosphorylase superfamily (PF01048.21)	No	Yes
OG0000013		(C)	S	NACHT (PF05729.13)	Yes	No
OG0000029	(C)		Q	Short-chain dehydrogenase (PF00106.26)	Yes	No
OG0000036	(C)		G	Glycoside hydrolase family 18 (PF00704.29)	Yes	Yes
OG0000041		(C)	G	Carboxylesterase family (PF00135.29)	Yes	No
OG0000049	(C)		D	Protein kinase domain (PF00069.26)	Yes	No
OG0000069		(C)	U	Trichothecene efflux pump (PF06609.14)	Yes	No
OG0000091	(C)		G	LysM domain (PF01476.21)	Yes	Yes
OG0000157		(C)	Q	Amine oxidase (PF01179.21)	Yes	No
OG0000187		(C)	U	Sugar transporter (PF00083.25)	Yes	No
OG0000321		(E)	Q	AMP-binding enzyme (PF00501.29)	Yes	No
OG0000702	(C)		E	Aminotransferase class III (PF00202.22)	Yes	No
OG0000852		(C)	S	Major facilitator family (PF07690.17)	Yes	No
OG0001175	(E)		K	Fungal specific transcription factor domain (PF04082.19)	Yes	No
OG0001226	(E)		IQ	AMP-binding enzyme (PF00501.29)	Yes	No

(C) and (E) indicate rapid contraction and expansion of each gene family, respectively

## Discussion

Since the global emergence of boxwood blight disease in the 1990s, research on the evolution of *Che* and *Cps* has focused primarily on understanding factors influencing pathogen population genetic and genomic diversity. However, gene family evolution in *Che* and *Cps* and its putative role in plant pathogenesis has not been studied. For this study, we identified and annotated rapidly evolving gene families in the genomes of *Che* and *Cps*, and 22 related fungal taxa representing taxonomic and trophic diversity in the family Nectriaceae to examine gene family contraction and expansion. Previous studies that have investigated gene family evolution in plant pathogenic fungi demonstrated that gene families important for pathogen-host interactions tend to expand rapidly [40, 48, 64]. Here, we tested the hypothesis that gene families important for plant host infection and pathogenesis have expanded in *Che* and *Cps*, relative to other pathogenic and saprobic species of *Calonectria* and distantly related non-*Calonectria* Buxaceae pathogens.

Among the pathogenic and saprobic species of *Calonectria* examined in this study, only *Che* and *Cps* exhibited predominant rapid contraction of gene families with putative involvement in host plant infection based on combined use of multiple annotation analyses. Gene families were assumed to play putative roles in plant pathogenesis based on examination of proteins that displayed similarity to proteins documented in the PHI database or contained predicted secreted effectors. While rapid expansion of gene families involved in pathogenesis has been reported for fungal plant pathogens, rapid contraction of pathogenesis-related gene families has been linked to biotrophy (obligate parasitism) and a restricted host range [3, 62, 65, 71, 78]. Given that infection by *Che* and *Cps* results in necrosis of diseased leaves and stems and because *Che* and *Cps* can be readily cultured on artificial nutrient medium, it is unlikely that they are obligate biotrophs. However, it remains unknown whether *Che* and *Cps* possess an initial biotrophic phase to obtain nutrients from living cells upon entry into plant tissues during infection, and whether these fungi exhibit hemibiotrophic or necrotrophic behaviors. Better studied fungal plant pathogens in the Nectriaceae including *Neonectria* spp. and *Fusarium* spp. have been characterized as hemibiotrophs [43, 57, 68]. Without knowledge of the trophic behavior and lifestyle of *Che* and *Cps*, it is challenging to interpret how the mechanism(s) of nutrient acquisition influence gene family evolution in these species. Future comprehensive investigation on the trophic behaviors and lifestyles of *Che* and *Cps* is warranted to provide additional insight into the relative contribution of these behaviors on contraction of gene families involved in plant pathogenesis in these species.

To date, *Cps* has been isolated and identified from environmental samples of diseased leaves on plants in three genera in the family Buxaceae: *Buxus*, *Pachysandra*, and *Sarcococca* [21]. This restricted host range provides at least one plausible explanation for predominant rapid contraction of pathogenesis-related gene families. *Che* causes disease primarily on species of *Buxus* in nature, although artificial inoculation experiments in the laboratory indicate that *Pachysandra terminalis* ‘var. Compacta’ is a host [4]. Differences in *Che* and *Cps* host range may explain observed differences in gene family evolution between these closely related species. Interestingly, contraction of gene families involved in trophic behavior and plant/animal host recognition among 45 fungal genomes sampled in the order Hypocreales were associated with a restricted host range [78]. Comparative genomic analyses of fungal insect pathogens in the *Ophiocordyceps unilateralis* species complex also revealed contraction of gene families involved in cuticle degradation and other insect-host interactions and specificity [71]. The contraction of gene families involved in cell-wall degradation and secondary metabolite biosynthesis associated with plant pathogenesis in fungal plant pathogens is also well documented [65]. In other plant pathogens, the absence or presence of pathogenicity-related genes have been shown to be strong determinants of plant host range for lineages of *Magnaporthe oryzae*,* Verticillium dahliae*,* Leptosphaeria maculans*—*Leptosphaeria biglobosa* species complex, and closely related *Zymoseptoria* species [12, 16, 25, 26]. For example, effector genes were hypothesized to have emerged after speciation and contributed to differences in host specificity in the closely related sister species *Zymoseptoria pseudotritici* and *Z. ardibiliae* [25]. Perhaps this is the case for *Che* and *Cps* and explains the limited overlap in rapidly evolving, pathogenesis-related gene families between these species. Based on these observations, our hypothesis that gene families involved in plant pathogenesis are expanding in *Che* and *Cps* was rejected.

Three gene families were rapidly evolving in both *Che* and *Cps*, but none had putative roles in plant pathogenesis. Instead, these gene families received COG annotations of unknown function, coenzyme transport, and cytoskeleton and did not contain proteins that received PHI annotations or secreted effector classifications. Individually, *Che* and *Cps* contained three rapidly evolving gene families each that were not rapidly evolving in any of the additional 22 fungal taxa investigated. In *Cps*, one of three uniquely evolving and rapidly contracting gene families contained a top Pfam target of CFEM domain (PF05730.12) which has a proposed role in fungal pathogenesis and contained putative secreted proteins [34]. In *Che*, none of the three uniquely evolving gene families had putative roles in pathogenesis. *Che* and *Cps* are closely related sister species with minor genetic and genomic differences, but exhibit phenotypic differences in thermotolerance, fungicide sensitivity, and secretome composition [24, 37, 74]. Additionally, the geographic distribution of *Cps* is more widespread than *Che* which has not expanded its range beyond the UK and continental Europe [38]. Differences in geographic range may contribute to the rapid evolution of different gene families in these species. However, the relationship between genetic diversity and biogeography are not well documented in fungal species except in certain pathogenic, mushroom-forming, and arbuscular mycorrhizal fungi [2, 18, 60, 79]. One isolate genome of *Che* and *Cps* was included for comparing rapidly evolving gene families between these species since pathogen populations of *Che* and *Cps* have been shown to be clonal in nature and display limited genetic diversity [37]. However, complete genome analyses of asexual pathogens like *Verticillium dahliae* have revealed that this plant pathogenic species can harbor substantial numbers of accessory genes, which can be enriched in candidate effectors not shared between strains [58]. Future studies should confirm similar trends in gene family evolution across genetically different isolates of *Che* and *Cps.*

Two of the closest known relatives of *Che* and *Cps* included in this study were the apathogenic soil saprobes, *Calonectria multiphialidica* (*Cmu*) and *C. naviculata* (*Cna*), which exhibited nearly exclusive rapid gene family expansion and had the greatest number of rapidly evolving gene families among *Calonectria* species investigated. The greatest number of rapidly expanded gene families in *Cmu* and *Cna* were classified into the unknown function, secondary metabolite biosynthesis, and carbohydrate metabolism COG categories. Expanded gene families involved in plant cell wall degradation and secondary metabolite biosynthesis are commonly observed in saprobic fungal genomes due to their involvement in nutrient degradation and defense against competing microorganisms, respectively [36, 39]. Mutualistic ecto- and endomycorrhizal fungi are also known to produce a variety of secreted PCWDEs and effectors known as mycorrhiza-induced small secreted proteins (MiSSPs) that allow them to initiate their symbiotic association with plants [45]. Interestingly, many rapidly expanding gene families in *Cmu* and *Cna* contained proteins similar to those characterized in pathogen-host interactions and were the same gene families rapidly contracting in *Che* and *Cps*. While there are no published reports of plant diseases caused by *Cmu* and *Cna*, rapid expansion of pathogenesis-related gene families suggests that these species may be evolving in a similar manner to plant pathogenic fungi [40, 48, 64]. Saprobic fungi have been shown to produce and secrete large repertoires of effectors similar in sequence to those produced by plant pathogenic fungi. However, the function of these effectors in a saprobic lifestyle remains unclear [22]. Functional annotation of gene families involved in pathogenesis in plant pathogenic *Calonectria* species of non-Buxaceae hosts, *Calonectria leucothoes* (*Cle*) and *Calonectria pseudoreteaudii* (*Cp*), were also performed in this study. *Cle* and *Cpr* are well documented pathogens of *Leucothoe* spp. and *Eucalyptus* spp. in the Ericaceae and Myrtaceae plant families, respectively [21]. Based on these reports, *Cle* and *Cpr* have a similar size and restricted host range to *Che* and *Cps.* However, the same observation of predominant rapid contraction of gene families involved in plant pathogenesis was not observed. Compared to *Che* and *Cps*,* Cle* and *Cpr* had fewer total rapidly evolving gene families and experienced predominant expansion of rapidly evolving gene families including those involved in plant pathogenesis. Based on these observations, *Cle* and *Cpr* displayed the most typical trends in gene family evolution observed in other fungal plant pathogens compared to the other *Calonectria* species examined [40, 48, 64]. A valuable and future comparison of gene family evolution between the restricted plant host range *Calonectria* species examined in this study and a species of *Calonectria* with a broader host range is warranted. At the time of initiating our study, relatively few *Calonectria* genomes were publicly available for this comparison. Lastly, we compared rapidly evolving gene families in *Che* and *Cps* to non-*Calonectria* Buxaceae pathogens *Pseudonectria buxi* (*Pbu*), *P. foliicola* (*Pfo*), and *Coccinonectria pachysandricola* (*Cpa*). *Pbu*,* Pfo*, and *Cpa* are the causal agents of Volutella leaf blight on different hosts in the family Buxaceae and are considered non-aggressive pathogens that typically occur on plants experiencing abiotic stress compared to *Che* and *Cps* [56, 73]. *Pbu* causes disease on species of *Buxus*, while *Pfo* causes disease on both *Buxus* and *Sarcococca* spp., and *Cpa* causes disease on *Sarcococca* and *Pachysandra* spp. [21]. *Pbu*,* Pfo*, and *Cpa* clustered with *Aquanectria penicillioides* and *Thelonectria rubi* with the fewest total rapidly evolving gene families while *Cpa* shared two rapidly contracting gene families involved in pathogenesis with *Che*. Because of the relatively low quantity of rapidly evolving gene families in *Pbu*,* Pfo*, and *Cpa* comparisons with *Che* and *Cps* were limited*.* However, the relatively low number of rapidly evolving gene families may suggest that these species are experiencing different selective pressures than *Che* and *Cps* [17].

In addition to the functional annotation analyses of pathogenesis-related gene families within *Calonectria* species and non-*Calonectria* Buxaceae pathogens, we performed broad functional annotation of gene families rapidly evolving across all 24 fungal taxa selected. Approximately, one third of rapidly evolving gene families across the 24 taxa were classified into the unknown function COG category. Within unknown function gene families, the predominant Pfam targets were heterokaryon incompatibility protein (HET; PF06985.12) and NACHT domain (PF05729.13). Both HET and NACHT domains are subunits of proteins commonly found in the *HNWD* gene family that allow fungi to recognize self from non-self for successful cell and cytoplasmic fusion [10]. Cell and cytoplasmic fusion are essential and fundamental processes in fungi that allows them to transition from unicellular to multicellular organisms and form hyphal networks for maximizing nutrient acquisition. HET genes have been shown to be highly polymorphic and contribute to the rapid evolution of members within *HNWD* gene families [10, 33, 53]. Constant rapid evolution of HET genes and their associated gene families allows fungi to maintain genome integrity and evade mycoparasitic exploitation or mycovirus infection, which is critical for fungal species success, irrespective of trophic behavior and lifestyle [52]. This would partially explain why the greatest number of rapidly evolving gene families contained proteins important for heterokaryon (vegetative) incompatibility across the 24 taxonomically diverse taxa examined in this study. Among species of *Calonectria*, heterokaryon incompatibility and HET loci have not been well studied. However, HET loci in *Che* and *Cps* likely have a similar function to other previously examined fungi in the Ascomycota where an incompatible (cell death) reaction is initiated when there are allelic differences at HET loci of two interacting fungal isolates of the same species.

## Conclusions

In this study, we used comparative phylogenomic methods to identify and characterize gene families that are rapidly evolving in *Che* and *Cps* and other closely related fungi to better understand adaptation and pathogenesis mechanisms for infection of hosts in the plant family Buxaceae by these pathogens. Our work highlights and provides new information on the evolutionary trajectories of *Che* and *Cps* and their close relatives that suggest a restricted host range in *Che* and *Cps* and gene family evolution trends in saprobic species *Cmu* and *Cna* that are analogous to many plant pathogenic fungi*.* Our results serve as a framework for future studies examining *Che* and *Cps* during infection and pathogenesis on Buxaceae hosts that may be used to develop novel disease management strategies. This research also raises new questions about the complex involvement of gene family evolution in the trophic lifestyles of *Calonectria* species and provides further evidence for an evolutionarily relevant role of pathogenesis-related gene families in fungi with saprobic lifestyles.

## Materials and methods

### Genome selection and assembly quality assessment

Twenty-four fungal taxa representing 10 genera in the family Nectriaceae and two outgroup species of *Stachybotrys* (Stachybotryaceae) were selected for this study (Additional file 1). Genome assemblies were obtained from NCBI GenBank for all taxa except *Calonectria multiphialidica*. References and accession numbers for genome assemblies are shown in Additional file 1. Genome data for *C. multiphialidica* were generated using Illumina sequencing technology and assembled as previously described [38]. Predicted protein sequence data were also downloaded from NCBI GenBank where available; otherwise, proteins were predicted using the Funannotate v1.8.1 pipeline [51]. Completeness of all predicted proteomes and underlying genome assemblies were assessed using BUSCO v3.1.0 using the fungal-specific gene set ‘Fungi odb9’ [63]. No plant material was used in this study.

### Estimation of gene families and construction of time-calibrated phylogeny

Clusters of orthologous genes were identified with OrthoFinder v2.2.7 using the “diamond” option for sequence alignment and “msa” option for gene-tree inference [20]. Single copy orthologs identified using OrthoFinder were concatenated into an alignment and poorly conserved regions were filtered with Gblocks v0.91 [6]. The best-fit model of protein evolution was identified using ProtTest v3.4.2 [14]. The protein sequence alignment was used to construct a maximum-likelihood phylogeny with RAxML v8.2.12 using the JTT model of protein evolution and 100 bootstrap replicates to assess confidence in tree topology [67]. The program r8s v1.81 was used to generate a time-calibrated ultrametric tree from the RAxML phylogeny using an estimated 244 MYA median divergence between *Stachybotrys chartarum* and *Fusarium graminearum* determined from the TimeTree database [35, 59]. The time-calibrated phylogeny and orthogroup data were used to measure changes in gene family size and identify rapidly evolving gene families.

### Identification and annotation of rapidly evolving gene families

Rapidly evolving gene families were identified using CAFE v4.2.1 which models gene family evolution through time using a stochastic birth and death model and identifies gene families that have experienced a significant change in size [28]. Input data were represented by the orthogroups (gene families) identified with OrthoFinder and the time-calibrated phylogeny representing evolutionary relationships among the 24 fungal taxa. A birth–death parameter (lambda, -s option) of 0.00353252 was estimated for the phylogeny using an optimization algorithm that maximizes the log likelihood of the data for all gene families. A default significance level of 0.01 (-p option) was used to calculate Viterbi *p*-values to assess rapid (significant) contraction or expansion of gene families along each branch. A custom Python script was developed to extract gene families that were rapidly evolving in extant species to perform additional analyses (Additional file 5).

Protein sequences within rapidly evolving gene families for each species were annotated to determine putative functional classes of gene families. Broad functional annotation was performed using eggNOG-mapper v2 which identifies Clusters of Orthologous Groups (COG) functional categories for each sequence using an e-value threshold of 1e−3 [30, 31]. The most frequently observed COG category within rapidly evolving gene families was used to categorize the gene families for further analysis. Gene families with equivalent frequencies of more than one COG category were classified to the first of the tied categories, which were ordered alphabetically for a given gene family. Protein sequences from *Aquanectria penicillioides* (20 total protein sequences) and *Pfo* (eight total protein sequences) did not receive COG annotations and were not used in gene family characterization. Additional annotation of protein sequences within gene families was performed by searching sequences against the Pfam-A database v33.1 using HMMER 3 with an e-value threshold of 1e−5 [19, 46]. For each annotation procedure, hits with the lowest e-value were selected for annotation of protein sequences that matched multiple subject sequences. Interspecific gene family annotation summaries and analyses were conducted in R v4.0.3 [55] using the following packages: tidyverse v1.3.0, UpSetR v1.4.0, ggtree v2.4.1, seqinr v4.2–5, Biostrings v2.58.0 [9, 11, 50, 72, 77].

### Identification of putative pathogenicity factors and secreted effectors

Protein sequences within rapidly evolving gene families from *Che*, *Cps*, two saprobic *Calonectria* species (*C. multiphialidica* [*Cmu*] and *C. naviculata* [*Cna*]), two *Calonectria* species pathogenic to non-boxwood hosts (*C. leucothoes* [*Cle*] and *C. pseudoreteaudii*, [*Cpr*]), and non-*Calonectria* pathogens of hosts in Buxaceae (*Pbu*, *Pfo*, and *Cpa*) were used to identify putative pathogenicity factors in the Pathogen Host Interactions (PHI) database v4.10 [70]. Searches were conducted using blastp v2.10.0 with an e-value threshold of 1e−5 [5]. Putative secreted proteins were identified (probability > 0.5) from protein sequences described above using SignalP v5.0 [1]. Predicted secreted proteins were further classified (probability > 0.5) as effectors using EffectorP v2.0 [66]. Unspecified parameters for all programs discussed were left as default values.

## Supplementary Information

**Additional file 1:** Genome metadata for the boxwood blight pathogens *Calonectria henricotiae* and *C. pseudonaviculata*, and 22 fungal taxa in the Nectriaceae.

**Additional file 2.** Maximum likelihood phylogenetic tree constructed from 2154 single copy orthologs that showed 100% confidence in tree topology for the boxwood blight pathogens *Calonectria henricotiae* and *C. pseudonaviculata*, and 22 fungal taxa in the Nectriaceae.

**Additional file 3.** Gene gains and losses identified by CAFE for rapidly evolving gene families in the boxwood blight pathogens *Calonectria henricotiae* and *C. pseudonaviculata*, and 22 fungal taxa in the Nectriaceae.

**Additional file 4.** Time-calibrated maximum likelihood phylogenetic tree constructed from 2154 single copy orthologs that showed 100% confidence in tree topology for the boxwood blight pathogens *Calonectria henricotiae* and *C. pseudonaviculata*, and 22 fungal taxa in the Nectriaceae.

**Additional file 5.** CAFE output statistics for the boxwood blight pathogens *Calonectria henricotiae* and *C. pseudonaviculata*, and 22 fungal taxa in the Nectriaceae.

**Additional file 6.** UpSet plots generated for comparison of rapidly evolving gene families in *Calonectria*, *Coccinonectria* and *Pseudonectria* species.

**Additional file 7.** COG and Pfam annotations and e-values for each protein sequence within rapidly evolving gene families of the boxwood blight pathogens *Calonectria henricotiae* and *C. pseudonaviculata*, and 22 fungal taxa in the Nectriaceae.

**Additional file 8.** PHI, SignalP, and EffectorP annotations for each protein sequence within rapidly evolving gene families in *Calonectria*, *Coccinonectria* and *Pseudonectria* species.

## Data Availability

The data generated or analyzed during this study are included in this published article and its supplementary materials (Additional file 1 through Additional file 8). NCBI GenBank accession numbers for genome assemblies generated or used in this study are as follows: *Aquanectria penicillioides* (GCA_003415625.1); *Calonectria henricotiae* (GCA_020623695.1); *Calonectria leucothoes* (GCA_002179835.1); *Calonectria multiphialidica* (GCA_020623665.1); *Calonectria naviculata* (GCA_003031705.1); *Calonectria pseudonaviculata* (GCA_020623675.1); *Calonectria pseudoreteaudii* (GCA_001879505.1); *Coccinonectria pachysandricola* (GCA_003693555.1); *Corinectria fuckeliana* (GCA_003385255.1); *Dactylonectria macrodidyma* (GCA_000935225.1); *Fusarium fujikuroi* (GCA_900079805.1); *Fusarium graminearum* (GCA_900044135.1); *Fusarium oxysporum* Fo47 (GCA_000271705.2); *Fusarium oxysporum* 4287 (GCA_000149955.2); *Fusarium solani* (GCA_000151355.1); *Ilyonectria destructans* (GCA_001913115.1); *Neonectria ditissima* (GCA_001305505.1); *Neonectria hederae* (GCA_003385265.1); *Neonectria punicea* (GCA_003385315.1); *Pseudonectria buxi* (GCA_003693515.1); *Pseudonectria foliicola* (GCA_003693505.1); *Stachybotrys chartarum* (GCA_000730325.1); *Stachybotrys chlorohalonata* (GCA_000732775.1); *Thelonectria rubi* (GCA_013420875.1).
