# Supplementary figures and images for: Comparative genomic analysis reveals contraction of gene families with putative roles in pathogenesis in the fungal boxwood pathogens Calonectria henricotiae and C. pseudonaviculata
Source: BMC Ecol Evol. 2022 Jun 21;22:79. doi: 10.1186/s12862-022-02035-4 (PMC9210730; doi:10.1186/s12862-022-02035-4)

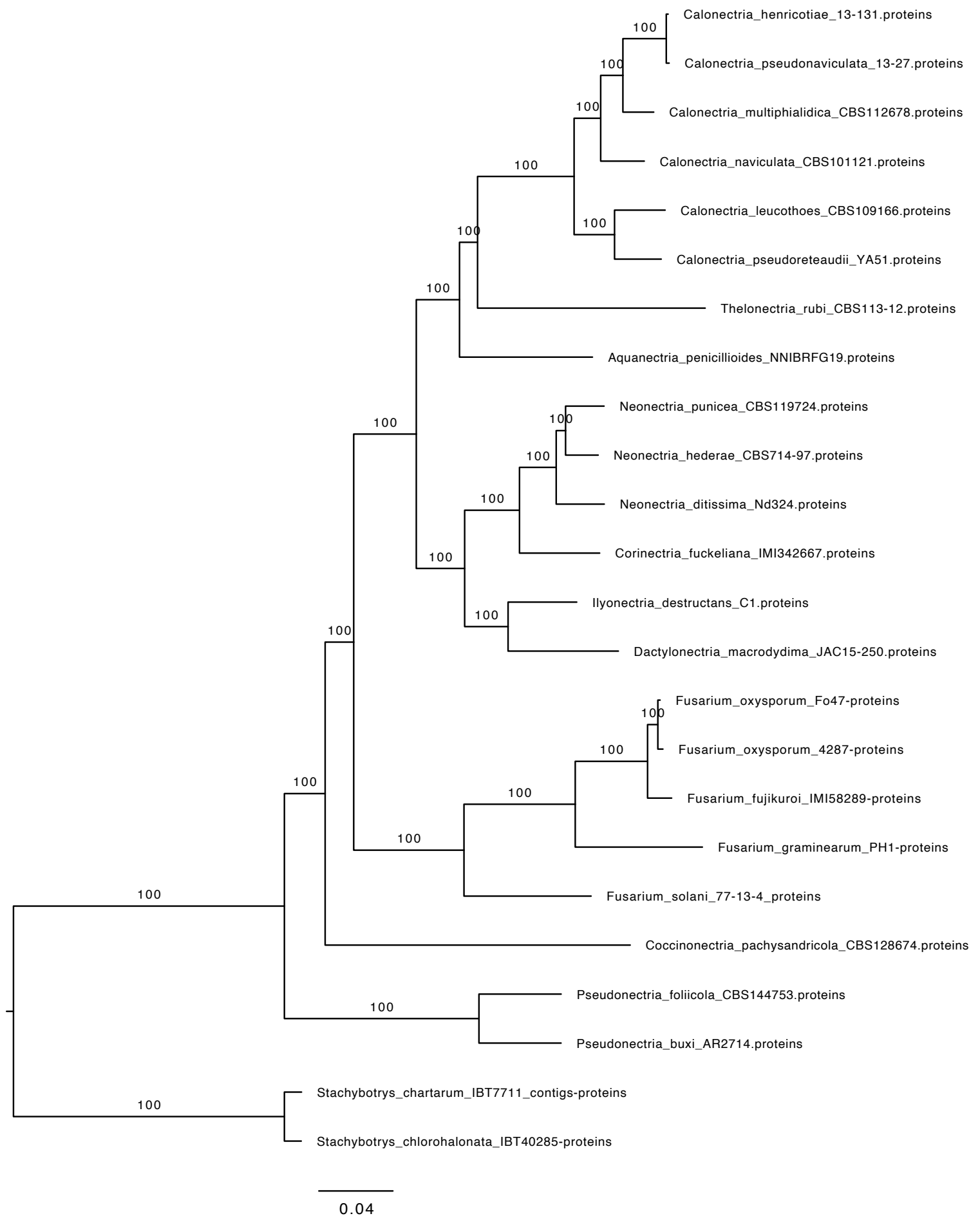

Supplement: Supplementary file 2 — Additional file 2. Maximum likelihood phylogenetic tree constructed from 2154 single copy orthologs that showed 100% confidence in tree topology for the boxwood blight pathogens Calonectria henricotiae and C. pseudonaviculata, and 22 fungal taxa in the Nectriaceae. [file 12862_2022_2035_MOESM2_ESM.pdf]
