# Supplementary material for: Comparative genomic analysis reveals contraction of gene families with putative roles in pathogenesis in the fungal boxwood pathogens Calonectria henricotiae and C. pseudonaviculata
Source: BMC Ecol Evol. 2022 Jun 21;22:79. doi: 10.1186/s12862-022-02035-4 (PMC9210730; doi:10.1186/s12862-022-02035-4)
Supplement: Supplementary file 6 — Additional file 6. UpSet plots generated for comparison of rapidly evolving gene families in Calonectria, Coccinonectria and Pseudonectria species. [file 12862_2022_2035_MOESM6_ESM.pdf]

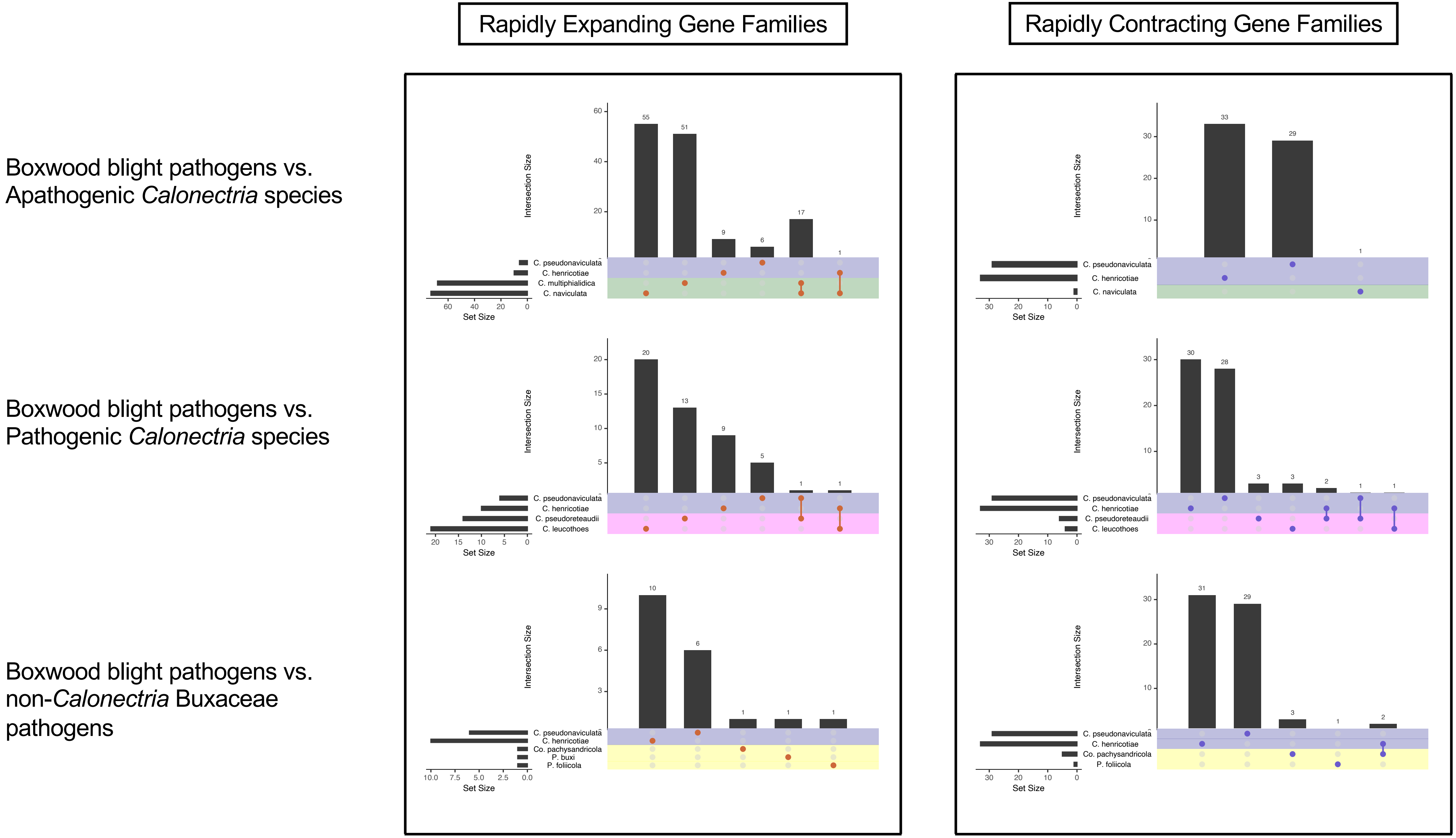


**Additional file 6.** UpSet plots generated for comparison of rapidly evolving gene families in *Calonectria, Coccinonectria* and *Pseudonectria* species.
